# Maximum power point tracking of photovoltaic module based on Particle Swarm Optimization enhanced with Quasi-Newton method

**DOI:** 10.1371/journal.pone.0327542

**Published:** 2025-07-10

**Authors:** Montaser Abdelsattar, Hamdi Ali Mohamed, Mohamed A. Ismeil, Ahmed A. Zaki Diab

**Affiliations:** 1 Electrical Engineering Department, Faculty of Engineering, South Valley University, Qena, Egypt; 2 Department of Electrical and Computers Engineering, El-Minia High Institute of Engineering and Technology, El-Minia, Egypt; 3 Electrical Engineering Department, Faculty of Engineering, King Khalid University, Abha, Saudi Arabia; 4 Electrical Engineering Department, Faculty of Engineering, Minia University, Minia, Egypt; PLOS, UNITED KINGDOM OF GREAT BRITAIN AND NORTHERN IRELAND

## Abstract

Maximum Power Point Tracking (MPPT) is a promising technology for extracting peak power from single or multiple solar modules for improving Photovoltaic (PV) system performance and satisfying economic operation. The tracker should continuously follow the MPP of the PV module at all operating and weather conditions. The Particle Swarm Optimization (PSO) algorithm represents a powerful optimal MPP tracker due to its simplicity and has enhanced greatest exploration characteristics. This article proposes a new technique based on PSO enhanced with Quasi-Newton local search for improving power quality while minimizing oscillation. This tracking process is making the MPPT comparable between high accuracy and fast tracking speed. MPPT proposal algorithm results are compared to the results of the hybrid PSO-P&O algorithm at different operating conditions. The proposed algorithm results show that MPP extraction has been done with a high-speed response and the best efficiency. Moreover, the PSO is enhanced with a Quasi-Newton (QN) local search method for tuning the optimal MPP.

## 1. Introduction

Photovoltaic (PV) systems are promising renewable energy sources for supporting the load demand of power distribution networks in recent years. They have many benefits, such as availability everywhere without transmission and generation costs, abundance, and environmental friendliness. However, they have faced two challenges. The first one is the lowering efficiency of PV modules, while the other challenge is the change in the amount of electrical power due to variations in weather conditions and non-linearity of the PV characteristics. Various factors such as temperature, humidity, wind speed, and dust influence the performance of PV systems. The dust and shading of sunlight spectra cause poor performance of PV panels [1–8]. The solar arrays must operate at a peak power point to improve their performance and efficiency. The Maximum Power Point (MPP) for each solar panel is only a single point on the I-V and P-V characteristic curves. This point requires special techniques called trackers to extract it to the PV system output. The Maximum Power Point Tracking (MPPT) maximizes the array output power at all operating conditions at different radiation levels and temperatures [5–9].

An electronic device should implement an MPPT algorithm to ensure the PV system operates permanently at MPP or close to it under all operating conditions. The MPPT algorithm should regulate the module’s voltage or current to achieve a stable Maximum Power despite the publication of numerous MPPTs in the literature, researchers continue to encounter obstacles in achieving optimal MPP tracking. There are many trackers in the literature that were still suffering from difficulties in reaching an optimal value [1**–**10]. The MPP tracker should typically extract the MPP regardless of weather or load conditions.

Owing to its algorithmic simplicity, ease of implementation, and effectiveness in addressing nonlinear optimization problems, conventional Particle Swarm Optimization (PSO) has been extensively employed in MPPT applications. Although PSO possesses many strengths, conventional PSO faces a challenge in the exact and efficient tracking of the MPP. The major challenges are susceptibility to steady-state oscillations, slower convergence rates, and propensity to become trapped in local optima in the context of rapidly changing weather conditions or partial shading scenarios. Inherently, the algorithm is random in updating particle velocities, which usually results in suboptimal performance, particularly in cases where fine-tuned local optimization is required. Due to these limitations, power losses occur, efficiency reduces, and tracking times become longer, necessitating the development of hybridized approaches that incorporate global exploration with fine-tuned local exploitation to mitigate these drawbacks.

### A. Literature review

Nowadays, MPPTs are classified into classical methods, optimal artificial intelligence algorithms, and hybrid algorithms, which are composed of either classical and optimal intelligence algorithms or two different artificial intelligence algorithms [10–29]. The classical MPPT algorithms in the literature were included in the Perturbation and Observation (P&O) algorithm and/or its modifications for enhanced tracking of MPP of PV systems at various weather conditions [2,16,10,23–26], Hill Climbing (HC) and its enhancement algorithms [27,28], Incremental Conductance (INC) [30,31] and others [32–37]. Most of these traditional methods still suffered from many drawbacks, such as instabilities and confusions of MPP as irradiance or load is changed rapidly or oscillated around MPP at steady-state, and also unguaranteed accuracy [11,38,39].

Artificial intelligence (AI) optimal methods [38–44] were proposed as MPPT by many researchers to overcome the drawbacks above, such as Fuzzy Logic Controller (FLC) [45–50], Artificial Neural Network (ANN) [51–53], PSO [54–57], PSO with Proportional Integrator (PSO-PI) [21]. An Arithmetic Optimal Algorithm (AOA) was implemented in a hybrid system combining PV and Thermoelectric Generation System (TEGS) to extract MPP at stochastic operating conditions [8]. Although these methods, such as MPPT, are effective, they require extensive computation and large data with a higher-cost processor [11,40]. The hybrid algorithms that are combined from classical and intelligence optimal algorithms, such as INC combined with optimal techniques [7,29], INC combined with Artificial Neural Network (INC-ANN) [14], P&O and PSO [58,17], P&O combined with FLC to suit dynamic weather conditions [13], hybrid tracker composed of P&O and Artificial Neural Network (PO-ANN) [14,18], P&O with PV Power Conditioning Controller (P&O-PVPC) [19]. Also, the hybrid optimal mathematical artificial algorithms were employed in the literature to extract MPP or Global Maximum Power Point (GMPP), such as the prediction tracker of the advanced Cuckoo Search (CS) [1], hybrid Gaussian process regression-Jaya (GPR-Jaya) [9], an adaptive neuro-fuzzy inference system-Particle Swarm Optimization (ANFIS-PSO) [22], three-point methods combined with FLC [12]. Among the MPP hybrid techniques, the hybrid algorithms that employed PSO seem to have high potential due to PSO being strong in construction, simple in operation, and less time in computation, and its ability to find MPP regardless of weather and load changes. These advantages make hybrid trackers that implement PSO represent powerful techniques for robustly tracking MPP in the field of social psychology. The PSO algorithm represents the easiest metaheuristic model to improve the MPPT capability and reduce MPP oscillation at steady-state [4,11,55].

Newton’s method involves a significant computational cost due to the Hessian matrix. The Quasi-Newton (QN) method reduces this cost by approximating the inverse Hessian matrix using first-order error function differentiation. This benefit is achieved through the Broyden-Fletcher-Goldfarb-Shanno (BFGS) algorithm [59–63].

Recent developments in MPPT optimization techniques have played a dominant role in advancing PV system performance. In order to optimize MPPT for urban PV systems under dynamic environmental conditions, hybrid adaptive-prediction methods are introduced that improve the tracking performance in comparison to both the standalone adaptive and prediction techniques [64]. Additionally, researchers demonstrate that innovative global MPPT algorithms with the capability to also solve such problems as partial shading are more efficient than the conventional approaches [65]. Additional advancements have been presented in the form of novel stochastic optimization techniques and improved neural network architectures for MPPT that provide additional robustness and precision in tracking [66–68]. These studies results demonstrated that hybrid MPPT algorithms that combine a global optimization method with a local optimization method are able to achieve faster convergence, higher accuracy, and smaller steady state oscillation.

QN is a numerical optimization method that deals with approximating the Hessian matrix of second derivatives that is expensive to calculate directly and is hence a practical method for large-scale optimization problems. It is the method used the most in the context of unconstrained optimization tasks, as it iteratively minimizes a given function. Curvature information of the objective function is incorporated in the search process in order to improve the convergence rate. The QN method used in this work is coupled with the global search of PSO to refine local solutions and enhance the accuracy of Maximum Power Point (MPP) tracking.

An iterative update of inverse Hessian approximation $B_{k}^{-1}$ is used by the QN method based on Broyden-Fletcher-Goldfarb-Shanno (BFGS) algorithm, which is one of the most efficient and popular QN techniques. The BFGS update is computed as follows:


$$B_{k+1}=B_{k}+\frac{y_{k}y_{k}^{T}}{y_{k}^{T}s_{k}}-\frac{B_{k}s_{k}s_{k}^{T}B_{k}}{s_{k}^{T}B_{k}s_{k}}$$ (1)

Where:

- $s_{k}=x_{k+1}-x_{k}$: the difference between successive iterations of the solution.
- $y_{k}=\nabla f\left(x_{k}+1\right)-\nabla f\left(x_{k}\right)$: the difference between successive gradients.
- $B_{k}$: the approximation of the inverse hessian matrix at the k−th iteration.

The initial approximation ($B_{0}=I$) is an identity matrix and we update iteratively. The QN method refines the search by moving in a direction $P_{k}$, as calculated by the following expression:


$$P_{k}=-B_{k}^{-1}\nabla f\left(x_{k}\right)$$ (2)

$\nabla f\left(x_{k}\right)$ is the gradient of the objective function at iteration k. Provided that the function value decreases enough, the step size $\alpha_{k}$ is determined by line search.

The local refinement is done on the global best solution ($g_{best}$) identified by PSO by integrating the QN method into the hybrid PSO algorithm. Equation (1) and Equation (2) provide iterative updates as described above, which help in providing the global exploration of PSO and the precise local exploitation of QN for the MPP tracking, thus increasing the accuracy as well as the speed of the MPP tracking.

### B. Research gap

As previously introduced, the review outlines various MPPT methods employed in the field, categorizing them into conventional, soft computing, and hybrid approaches. researchers and practitioners commonly employ conventional techniques such as P&O, INC, and others, but their effectiveness is limited, particularly in the face of rapid weather variations. They struggle to efficiently observe or track the MPP, often resulting in long tracking times, low accuracy, and significant oscillations.

In contrast, soft computing methods, such as ANN, FLC, PSO, Genetic Algorithm (GA), and several others, offer robustness and flexibility, leveraging AI and evolutionary principles. They excel in handling non-linear tasks, providing superior tracking performance compared to conventional methods.

Hybrid MPPT methods have emerged as a solution to address the limitations inherent in singular approaches. These hybrids can combine two conventional methods: a hybrid soft computing-conventional algorithm or two soft computing algorithms. They aim to improve tracking performance by addressing the drawbacks of individual methods.

### C. Paper contribution

In the same aspect of hybrid PSO algorithms, this research work investigates the implementation of hybrid PSO with a QN local search method [55,56] for tuning the MPP. The tracking algorithm is based on the swarm intelligence theory. PSO produces the nearest optimal MPP value, and QN is used to fine-tune precise MPP with minimum steady-state oscillations. A two-diode module model is implemented to produce peak power points at different weather and load conditions. Hybrid Practical Swarm Optimization (H-PSO) with QN for tuning the local search algorithm can extract the MPP with a fast response and minimum steady-state power oscillations around MPP.

**This paper presents several key contributions as follows:**

- A hybrid maximum power point algorithm is introduced, based on the hybridization of the PSO algorithm and the QN local search method.
- The hybrid algorithm is designed to combine the exploratory features of PSO with the refined exploitation characteristics of QN, strategically incorporating existing solutions within the genetic algorithm framework.
- A comprehensive comparative analysis is conducted between the proposed method and the conventional PSO technique.
- The effectiveness of the hybrid algorithm is validated through rigorous testing on the MATLAB/Simulink simulation platform.

### D. Paper organization

This paper divides into the following main sections: Section II focuses on PV modeling, detailing the methodologies employed. In Section III, the paper delves into the practical implementation of the H-PSO method. Following this, Section IV encompasses system simulations and presents the obtained results. Finally, Section V summarizes the study’s conclusion.

## 2. PV Modelling

**Fig 1:**
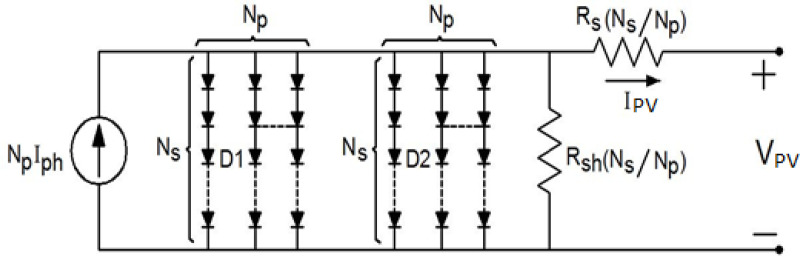
Two-diode module model.


Fig 1 shows a two-diode module model. The model equation is written as follows [57,59]:

$$I_{PV}=N_{p}I_{ph}-N_{p}I_{s1}\left\{\exp\left(\frac{V_{PV}+I_{PV}R_{s}\left(\frac{N_{s}}{N_{p}}\right)}{a_{1}.N_{s}.V_{T}}\right)-1\right\}-N_{p}I_{s2}\left\{\exp\left(\frac{V_{PV}+I_{PV}R_{s}\left(\frac{N_{s}}{N_{p}}\right)}{a_{2}.N_{s}.V_{T}}\right)-1\right\}-\left(\frac{V_{PV}+I_{\text{PV }}R_{s}\left(\frac{N_{s}}{N_{p}}\right)}{R_{\text{sh }}\left(\frac{N_{s}}{N_{p}}\right)}\right)$$ (3)

$$I_{\text{ph }}=\left(G/G_{STC}\right)\left[I_{\text{ph at STC}}+K_{i}\left(T-T_{STC}\right)\right]$$ (4)

$$I_{s1}=I_{s1\text{ at STC}}{\left(\frac{T}{T_{STC}}\right)}^{3}\exp\left[\left(\frac{q.E_{g}}{a_{1}.K}\right)\left(\frac{1}{T_{STC}}-\frac{1}{T}\right)\right]$$ (5)

$$I_{s2}=I_{s2\text{ at STC}}{\left(\frac{T}{T_{STC}}\right)}^{3}\exp\left[\left(\frac{q.E_{g}}{a_{2}.K}\right)\left(\frac{1}{T_{STC}}-\frac{1}{T}\right)\right]$$ (6)

where:

I_ph_ is the module-generated current,

N_p_ is the No. of parallel modules,

N_s_ is the No. of series modules,

R_s_ is the series resistance of the array,

R_sh_ is the shunt resistance of the array,

V_T_ is the thermal voltage of the array,

a_1_ and a_2_ are ideality factors for D1 and D2 respectively,

I_pv_ is the output current of the array,

V_pv_ is the output voltage of the array,

I_s1_ is the saturated current of the array,

I_s2_ is the recombination current of the array,

G is the irradiation level,

G_STC_ is the irradiation intensity at standard test conditions (STC),

T is the operating temperature of the array,

T_STC_ is the temperature of the array at STC,

I_ph_ at STC is the generated current at STC,

K_i_ is the factor of short circuit current,

K is the constant of Boltzmann (1.38*10^-23^ J/kelvin),

q is the charge of the electron (1.6*10^-19^ C), and

E_g_ is the energy gap of silicon.

## 3. Hybrid practical swarm optimization (H-PSO)

### A. Particle Swarm optimization (PSO)

The PSO approach is a promising optimal methodology that was developed by Eberhart and Kennedy in 1995. It has a robust solution for non-linear and non-differentiating problems based on the social-psychological [54–57]. It was based on the bird’s behavior to find its finest location in the swarm. PSO has become famous nowadays due to its simplicity and ease of implementation [54–57].

In PSO, every solution is a particle that has position and velocity. The particle groups are forming a swarm in a multidimensional space. Normally, each particle has the ability to remember its finest position and to know its global position. Swarm particles have good contact with each other to adjust their positions and velocities with respect to the finest positions. The PSO equations can be obtained as follows [54–57].


$$v(k+1{)}_{i,j}=w.v(k{)}_{i,j}+c_{1}r_{1}(\text{gbest}-x(k{)}_{i,j})+c_{2}r_{2}({\text{pbest}}_{j}-x(k{)}_{i,j})$$ (7)

$$x(k+1{)}_{i,j}=x(k{)}_{i,j}+v(k{)}_{i,j}$$ (8)

where:

$v_{i,j}$: Particle i velocity at dimension j,

$x_{i,j}$: Particle i location at j dimension,

c_1_, c_2_: they are factors of accelerator,

w: it is the constant of inertia weight,

r_1_, r_2_: numbers have random values between 0 and 1,

pbest: the position which has the finest location of a specific particle,

gbest: finest particle location of the group.

**The algorithm can be optimized with PSO as follows:**

1- *Set particle and search parameter numbers for position and velocity.*
2- *The location and speed of each particle are randomly initiated.*
3- *Every particle value is computed for fitness.*
4- *The Gbest (Global Finest) for particle.*
5- *Update particle location and speed referring to the Gbest.*
6- *Step 3 & 4 are repeated up to optimal value.*
7- *Gbest is the optimal value at the final iteration.*
8- *Estimate the optimal case of power.*
9- *Fed the optimal particle position of the duty cycle.*

### B. Combining PSO with the Quasi-Newton method

The results of the PSO can be updated by sharing other computation techniques. Many authors used Differential Evolution (DE) with the PSO algorithm. The diversity of the population is increased by 1) either stopping the particles’ motion from being too close to each other and colliding or 2) auto-adapted parameters such as the building factor, speeding-up factors, or inertia weight.

Newton’s method is a computational cost for the Hessian matrix. The QN method approximates the inverse Hessian matrix to error function differentiation from the first order by using the Broyden-Fletcher-Goldfarb-Shanno (BFGS) approach [60–63]. QN method and PSO algorithms complement each other. In this proposed algorithm, the PSO finest solution represents an initial parameter for the QN method.

### C. Hybridization enhancement

The hybrid PSO algorithm integrates the strengths of PSO with a QN local search method to improve optimization performance. Here’s a detailed explanation:

PSO is a nature-inspired optimization technique that models the behavior of bird flocking or fish schooling. In PSO, a population of potential solutions, known as particles, moves through the search space. Moreover, each particle adjusts its position based on its velocity and the finest position found by itself and its neighboring particles. In addition, particles are influenced by their own experience (finest solution found) and the collective behavior of the swarm.

On the other hand, QN methods are iterative optimization techniques used to find the minimum of a function. They approximate the Hessian matrix, which represents the second derivatives of the objective function, without directly computing them. This approach provides an estimation of the curvature of the function’s surface. The hybridization process combines the PSO algorithm with the QN local search method to leverage their individual advantages. While the PSO is excellent for global exploration due to its swarm behavior, the QN methods are effective in local exploitation by approximating the curvature of the objective function. So, by integrating the QN local search, the hybrid algorithm performs more sophisticated local exploitation around promising regions identified by the PSO. Moreover, the hybrid PSO-QN improves the convergence characteristics as follows: PSO might struggle with local optima in certain complex landscapes. The local search capability of QN methods helps in converging to optimal or near-optimal solutions efficiently.

Implementation of the Hybrid PSO algorithm with QN Local Search:

- Initialization: Initialize particles’ positions and velocities randomly within the search space.
- PSO Movement: Each particle updates its position based on its velocity and the best positions found by itself and its neighbors in the swarm.
- Local Search Incorporation: At specific intervals or under certain conditions, the algorithm applies the QN method to refine the position of selected particles.
- Iterative Refinement: The process iterates, allowing particles to explore the search space using PSO and refine their positions alternately using the local search method.
- Termination Criterion: The algorithm stops when a stopping criterion is met, such as a maximum number of iterations or convergence within a tolerance threshold.

The advantages of Hybrid PSO with QN can be summarized as the following:

- Improved convergence speed by exploiting the local search capability.
- Enhanced exploration of the search space through the PSO’s global exploration.
- Effective handling of multimodal optimization problems with both global and local search capabilities.

In summary, the hybridization of PSO with the QN local search method combines global exploration and local exploitation to navigate complex optimization landscapes efficiently, resulting in improved convergence toward optimal solutions. In the same regard, applying the hybrid PSO with QN enhances the efficiency of the MPPT tracker in finding the best global MPP. It enhances the performance of the tracker to reduce the oscillation around the MPP.

### D. Flowchart of proposed algorithm

**Fig 2:**
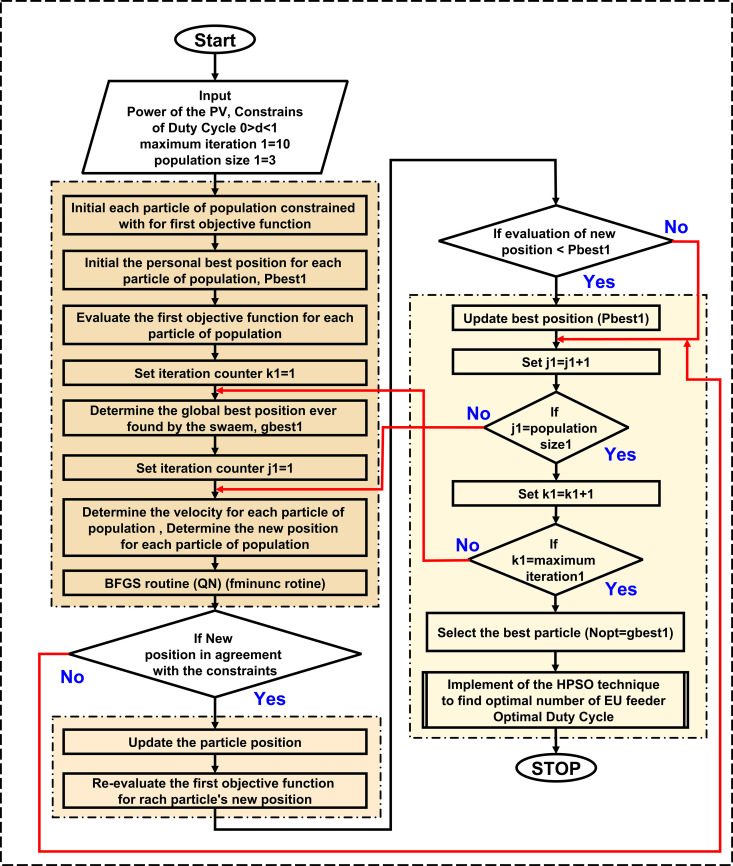
Flowchart of H-PSO algorithm.

Fig 2 demonstrates the flowchart of the H-PSO algorithm for extracting the MPP of the PV system. This proposed algorithm has powerful tools for both PSO and QN.

## 4. Simulation and results of PV system

**Fig 3:**
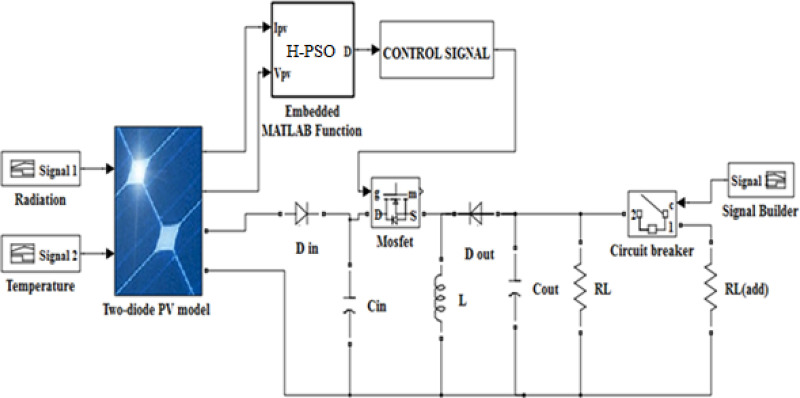
MATLAB–Simulink model of MPPT based on hybrid PSO technique.

Fig 3 contains the MATLAB/ Simulink approach of the solar array. The solar model is constructed from PV multi-crystalline modules, a proposed hybrid PSO algorithm, a DC/DC buck-boost converter, and an RL.

The results of the H-PSO algorithm are done for the following case studies:

A Change of solar radiation
B load change
C Temperature variation

The PV model consists of four series modules which are types of MSX-60. The module-rated power is 60 W to produce 240 W for the PV model. (MSX-60) module and DC/DC buck-boost converter parameters are given in Table 1 [11,59].

**Table 1:** MSX-60 and DC/DC buck-boost converter Data.

Solar cell module parameters				Data of buck-boost converter	
Module parameter	Data Value [59]	Module model parameter	Data results [59]	Converter parameter	Parameter value [11]
${𝐏}_{mp},𝐖$	60.000	${𝐈}_{ph},𝐀$	3.8084	𝐋,𝐇	1.000x10^-3^
${𝐈}_{sc}$, A	3.8000	${𝐈}_{𝐬1},𝐀$	4.8723*10-10	${𝐂}_{in},𝐅$	470.0000x10^-6^
${𝐕}_{oc},𝐕$	21.1000	${𝐈}_{𝐬2},𝐀$	6.1528*10-10	${𝐂}_{out},𝐅$	220.0000x10^-6^
${𝐈}_{mp},𝐀$	3.5000	${𝐑}_{𝐬},\Omega$	0.3692	f,Hz	50.000x10^3^
${𝐕}_{mp},𝐕$	17.1000	${𝐑}_{sh},\Omega$	169.0471	${𝐑}_{𝐋},\Omega$	50
${𝐊}_{𝐯},𝐕/𝐂\circ$	-80.0000*10-3	${𝐚}_{1}$	1.0003		
${𝐊}_{𝐢},𝐀/𝐂\circ$	3.000*10-3	${𝐚}_{2}$	1.9997		
${𝐍}_{𝐬}$	36.000				
**N**_**p**_	1.000				

The choice of the modified PSO and Hybrid PSO algorithms for comparison arose from their applicability in handling the main disadvantages of conventional MPPT techniques. PSO-based algorithms are well known for their flexibility and efficiency in different conditions, preferable for non-linear MPPT problems. P&O and INC were not used for the following reasons: they are representative of classical methods with notorious drawbacks, including large-amplitude oscillations and unsatisfactory stability in conditions of high-frequency environment variations. The hybrid PSO algorithm was presented for addressing the conventional limitations associated with PSO’s slow convergence speed and local optima inclusion by incorporating the QN technique to augment precision and parsimony. Focusing on these two algorithms was done to provide a more detailed and exhaustive assessment while acknowledging that widening the comparison would be computationally intensive and could include methods that add noise to the evaluation but do not introduce a significant improvement in practice.

### A. Change of solar radiation

**Fig 4:**
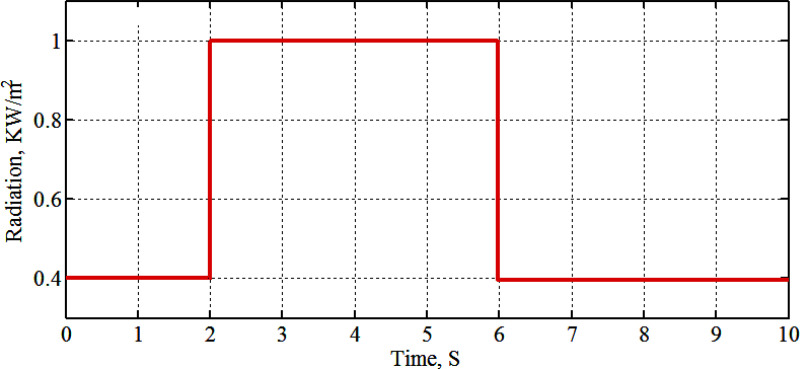
Variation of solar irradiance.

**Fig 5:**
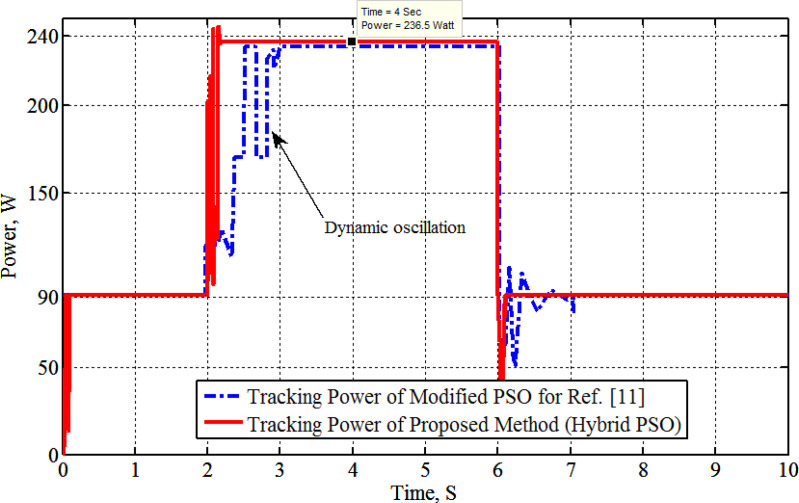
PV tracking power during irradiance variation.

**Fig 6:**
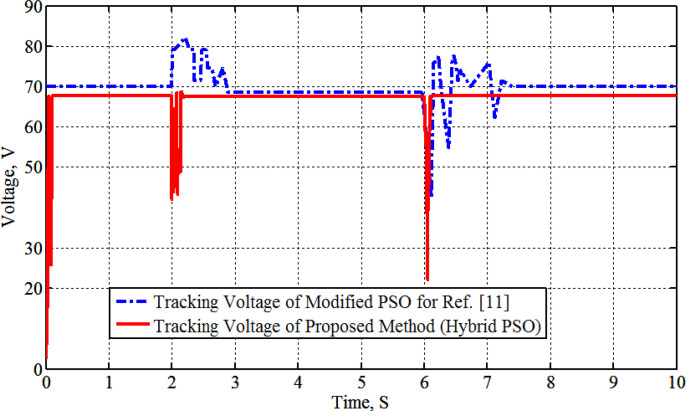
PV tracking voltage at various radiation.

**Fig 7:**
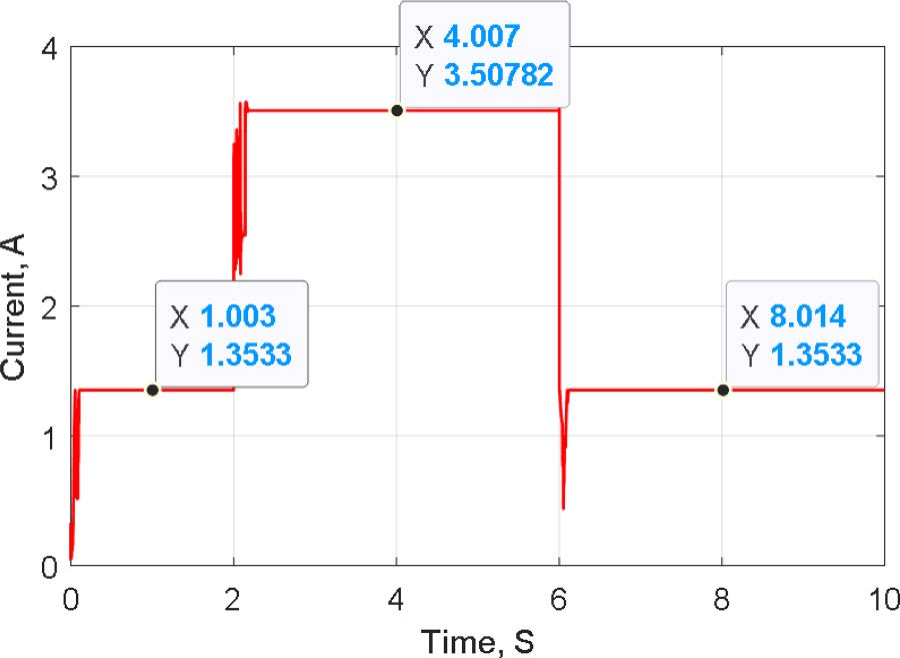
PV tracking current at various radiation using HPSO.

**Fig 8:**
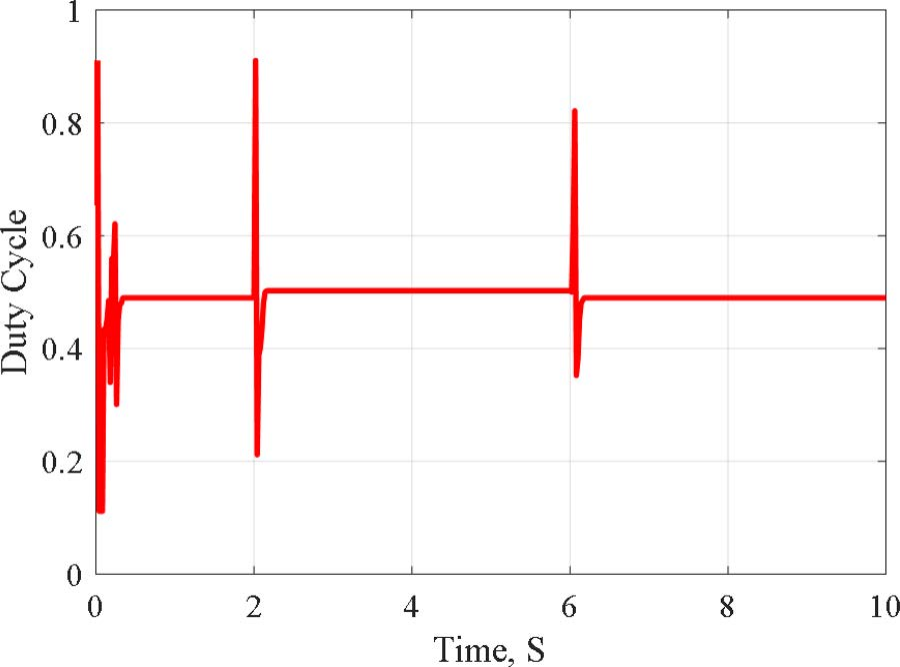
Duty Cycle at various radiation using HPSO.


Fig 4 shows the graph of solar radiation, which at 2s jumped up from 0.4 kW/m^2^ to 1.0 kW/m^2^ and stills at this level until 6s at which is abruptly changed down to 0.4 kW/m^2^ at 25°C. The maximum generated power with its associated voltage and current, as compared with the PSO-P&O results of Ref. [11], due to the solar radiation graph of Fig 4. The results are shown in Fig 5, Fig 6, Fig 7 and Fig 8. These figures show that the H-PSO algorithm has more accuracy and ability than the PSO-P&O algorithm of Ref. [11]. The tracking power efficiency is evaluated as follows:

η= PT/ PC (9)

where:

PT is the tracking power of PV modules.

PC is the rated power of the PV modules.

Fig 5 indicates that the PT of modules (MSX-60) =236.5W and the tracker efficiency = (236.5/240)*100 = 98.6%.


PC=module power * modules No.=60*4=240W

The results show that the H-PSO proposed tracker has a high efficiency of (98.6%) without minimum oscillation, which enhances the hybridization with the QN local search method.

Moreover, Fig 5 shows that the H-PSO reached the MPP in less time than the compared algorithm. The H-PSO algorithm reached the MPP after 0.2 seconds, while the compared algorithm reached it after 1 second. Moreover, the characteristics of the system with the H-PSO have been shown in Fig 6 for the voltage of the PV. While Fig 7 shows the PV current at various radiation using H-PSO. Moreover, Fig 8 shows the duty cycle at various radiation using HPSO.

### B. Temperature variation

**Fig 9:**
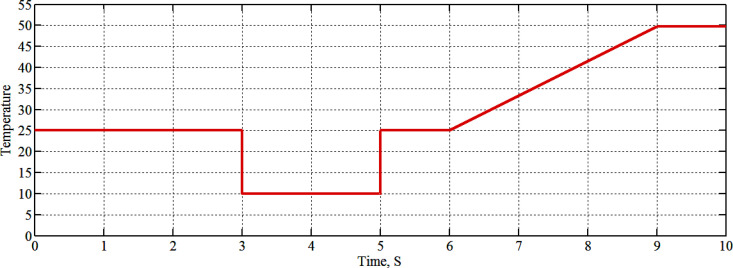
The curve of temperature changing.

The case study is for temperature variation and is not found in Ref. [11] for comparing the results. The four series modules are operated at the temperature graph as shown in Fig 9 at solar radiation of 1000W/m^2^ as follows:

The graph of the module’s temperature starts at 25°C and steps down to 10°C at a time between 3–5 seconds and then jumps high to 25°C, linearly increasing up to 50°C at the time of 9 seconds, and the temperature graph stays at 50°C between the time of 9–10 seconds as demonstrated by Fig 9.

**Fig 10:**
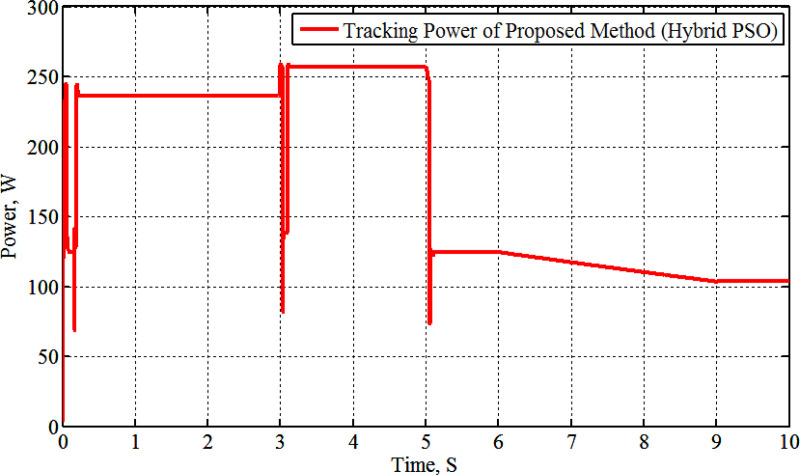
Tracking PV power during temperature variation.

Fig 10 illustrates the results of the solar power approach. This figure illustrates that the proposed H-PSO algorithm is accurate and powerful for MPP tracking according to temperature variation. The interpretations have been occurring during the load change as a result of the algorithm’s randomization to reach the MPP.

**Fig 11:**
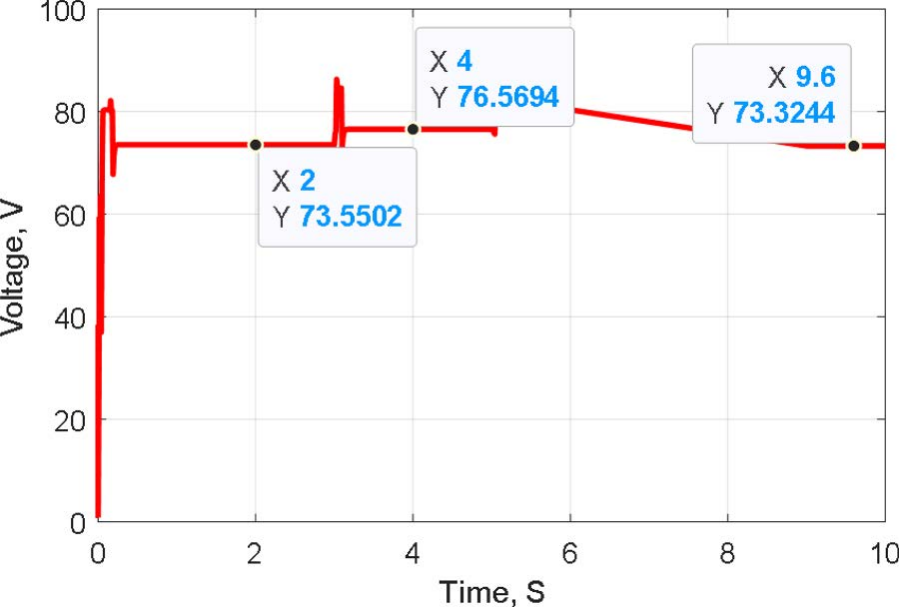
Tracking PV Voltage during temperature variation.

**Fig 12:**
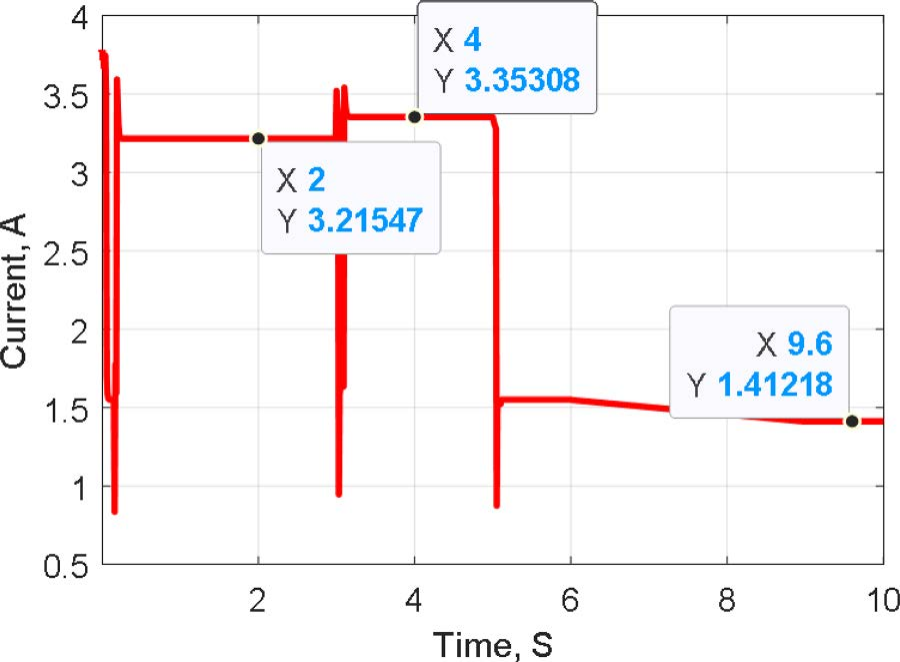
Tracking PV Current during temperature variation.

**Fig 13:**
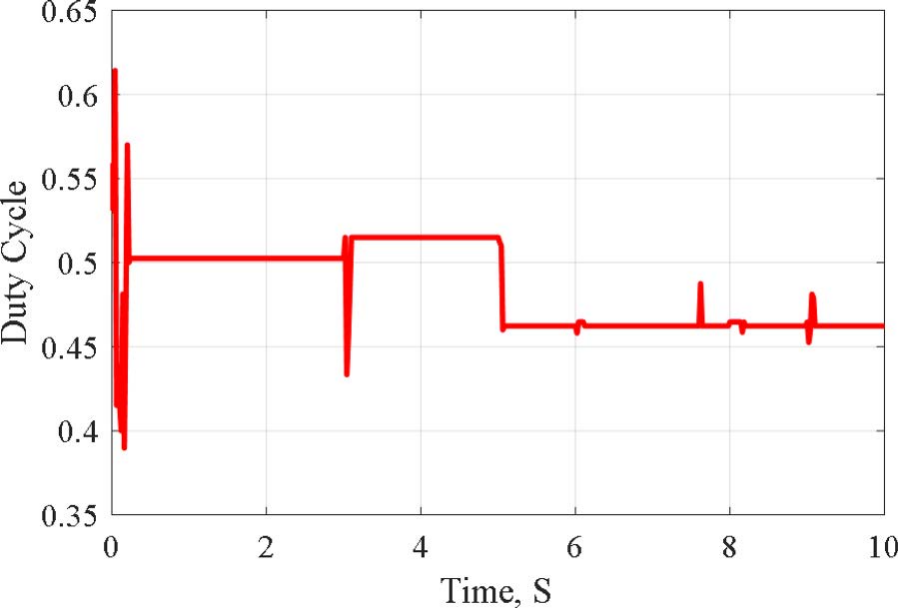
Duty Cycle during temperature variation.

Figs 11–13 illustrate the system’s response to temperature variations. Fig 11 shows the tracking of PV voltage, demonstrating how the system adjusts to maintain stable operation. Fig 12 presents the corresponding PV current variations, reflecting changes in power generation due to temperature fluctuations. Fig 13 depicts the duty cycle adjustments, highlighting the controller’s role in optimizing performance under dynamic conditions.

PV systems have gained significant attention as a key renewable energy source, with advancements in MPPT techniques being crucial to enhancing their efficiency. Various research efforts have been made to optimize the performance of PV systems through the application of advanced algorithms. In the study [69], artificial intelligence was integrated to enhance grid-connected PV systems, while the work presented in [70] focused on single-phase inverters with MPPT for residential applications. In addition, according to the research in [71,72], machine learning techniques and image processing were explored for fault detection and energy prediction in PV systems, highlighting their potential in improving reliability and efficiency. Furthermore, economic and environmental aspects of renewable energy sources, including wind and hybrid systems, are discussed in [73] and [74], underscoring the importance of sustainable energy management. Studies such as [75,76] have also examined microgrid energy management and the integration of PV systems with compensators. Finally, research into the negative effects and mitigation strategies for renewable energy in power systems provides additional insights into optimizing system performance [77]. These contributions collectively inform the development of advanced MPPT techniques, such as PSO enhanced with QN methods, to improve the efficiency and stability of PV systems. Numerous research efforts have explored various aspects of PV technology, including efficiency improvements, fault detection, and energy forecasting, further reinforcing the significance of ongoing advancements in this field [78,79].

### C. Load change

**Fig 14:**
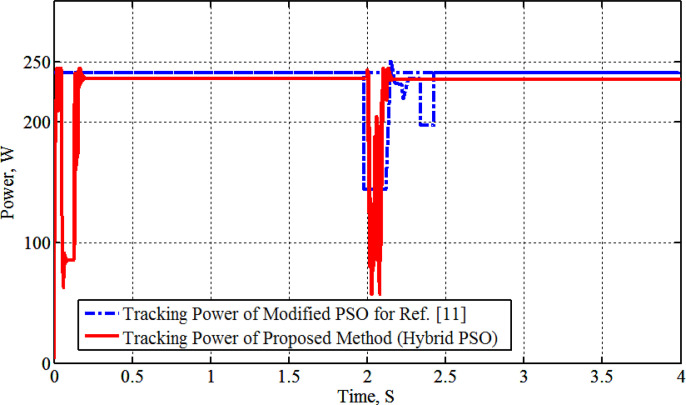
Tracking PV power at load increasing.

**Fig 15:**
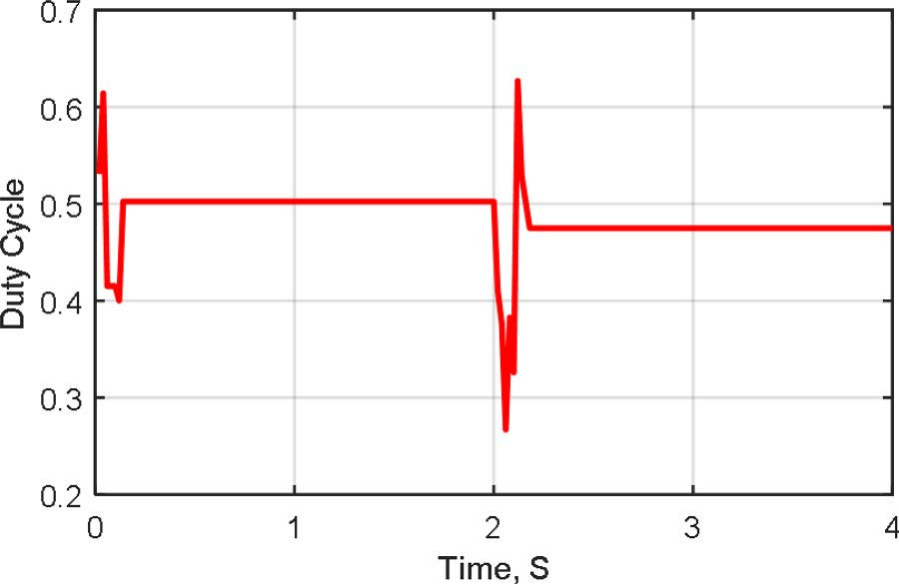
Duty Cycle at load increasing.

Fig 14 shows two curves of optimal power tracking by two trackers of both the proposed H-PSO and PSO-P&O of Ref. [11] during the load change. The output optimal tracking power has a value of 240W at 1000 W/m^2^ of irradiance level and 25°C of temperature value. The tracking power graph indicates a sudden 50% load increase at 2 seconds. This is done by connecting a resistor of 100 Ω in parallel with a load resistor of 50Ω. The increasing load causes a sudden decrease in tracking power due to the effect of load balance and transition. The H-PSO tracker then swiftly reacts to restore power generation with minimal oscillation, in contrast to the PSO-P&O results presented in Ref. [11]. On the contrary, interpretations have arisen amidst the load changes due to the algorithm’s randomized nature in reaching the MPP. Moreover, Fig 15 illustrates the duty cycle operation in this case study, highlighting the system’s response to load variations.

### d. Performance under rapidly changing shading conditions

MPPT algorithms face significant challenges in dynamic shading conditions, which produce a complex P-V curve with multiple local maxima and thus varying locations over time. The GMPP is moving, which conventional algorithms tend to track inefficiently. In order to address this issue, a new Hybrid Particle Swarm Optimization (H-PSO) with Quasi-Newton (H-PSO) is proposed. The global exploration capability of PSO allows PSO to traverse several local maxima to find the GMPP even under dynamic conditions. The QN method is included to allow for the precise local refinement of the solution to prevent oscillations and to speed up convergence. The proposed H-PSO algorithm is able to track the GMPP with average efficiency higher than 98.5% under rapidly varying shading profiles, while compared with traditional methods such as PSO-P&O, it is shown to have faster convergence and less oscillation around the power point. Based on real-time P-V curve updates, algorithm dynamically adjusts particle positions and velocities to continuously adapt to shading changes. Additionally, the Quasi Newton method, when applied periodically, improves the precision of the local search, and the algorithm is able to converge to the true GMPP very quickly with minimum power loss. The hybridized approach of this work guarantees not only the robustness of tracking processes under challenging conditions, but also improves the reliability and efficiency of PV systems.

### e. Computational resource analysis of MPPT algorithms

In Table 2, a comparative analysis is made based on computational complexity, convergence speed, and resource requirements of various MPPT algorithms summarized in terms of computational resource requirements. The complexity and resource demand of conventional PSO are only moderate, but its convergence speed is only moderate. Although more complicated and resource-consuming, PSO-P&O has comparable convergence characteristics and therefore is a balanced option. Although the PSO Enhanced with QN Method has higher computational complexity and resource requirements stemming from gradient calculations, it improves the convergence speed considerably and thus is appropriate for dynamic conditions in which fast response is essential. On the other hand, Low-Complexity PSO reduces computational requirements and resource usage at the cost of varying convergence speed from one design to another and is more suitable for applications with limited computational power.

**Table 2:** Computational resource comparison of MPPT algorithms.

Algorithm	Computational Complexity	Convergence Speed	Resource Requirements
Conventional PSO	Moderate	Moderate	Moderate
PSO-P&O	Slightly Higher	Moderate	Slightly Higher
PSO Enhanced with QN Method	Higher	Faster	Higher
Low-Complexity PSO	Lower	Moderate or Slower (depending on design)	Lower

### f. Key findings

The results show that the proposed Hybrid PSO with Quasi-Newton (H-PSO) method works better than the traditional PSO based methods in tracking Maximum Power Point (MPP). We also find a significant reduction in the oscillation around the MPP, faster convergence times, as well as better tracking efficiency under different operating conditions like irradiance, load and temperature changes. In the irradiance variation case, the H-PSO method achieves a remarkable tracking efficiency of 98.6%, which demonstrates that H-PSO can operate close to the theoretical maximum power output. In addition, H-PSO responds very quickly, with MPP tracked within 0.2 seconds, while MPP tracking by PSO-P&O takes 1 second, which illustrates that H-PSO is effective in dynamic environments. The hybridization of global exploration (PSO) with local refinement (QN) optimized the exploration vs. exploitation tradeoff and resulted in these improvements. In addition, the robustness of H-PSO was demonstrated for different scenarios, and its reliable performance was still shown during abrupt changes in load and temperature. This research shows that H-PSO can be a promising way to increase the efficiency and stability of PV systems and sets a benchmark for other MPPT algorithms.

**Fig 16:**
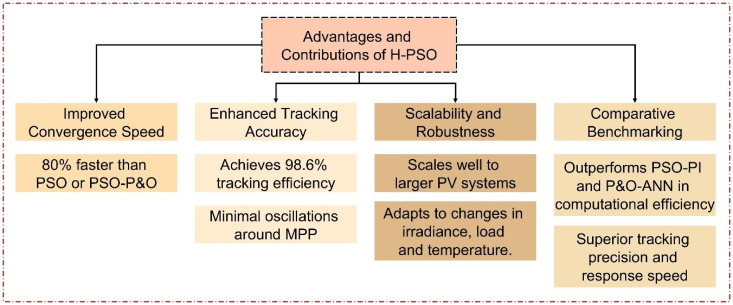
Key advantages of the proposed H-PSO method.

Fig 16 illustrates the benefits of the proposed H-PSO approach, which include accelerated convergence, 98.6% tracking accuracy, resilience under dynamic situations, and enhanced efficiency relative to PSO-P&O and PSO-ANN.

The QN method is integrated with the PSO algorithm to improve its ability to find global maximum peaks by improving the local search process. PSO has great global exploration ability using swarm intelligence to explore broad solution spaces, but it is prone to premature convergence and local optima when the problem is complex or dynamic. The QN method is well known for its iterative refinement and efficiency in approximating curvature using the Broyden-Fletcher-Goldfarb-Shanno (BFGS) approach and complements PSO by supplying precise local exploitation. After PSO detects a promising region (global best position), the QN method is used to refine the solution by using second-order gradient approximations to speed up convergence and guarantee accurate peak identification. This hybrid approach both damps steady-state oscillations and guarantees that the global maximum peak is tracked effectively under rapidly varying irradiance or partial shading conditions.

## 5. Conclusion

This paper presents a novel H-PSO algorithm for efficiently extracting the Maximum Power Point (MPP) of PV systems. The H-PSO algorithm demonstrates high-speed tracking with minimal steady-state oscillations, owing to the integration of the QN local search method, which enhances the global search capabilities of PSO. The proposed H-PSO tracker achieves a tracking efficiency of 98.6% under varying irradiance levels, significantly higher than the comparative PSO-P&O algorithm. Additionally, the H-PSO algorithm reaches the MPP in 0.2 seconds, which is 80% faster compared to the PSO-P&O algorithm that requires 1 second to achieve the same. Power oscillation around the MPP is minimized to a negligible level, ensuring stable performance even under dynamic weather and load conditions. Under irradiance variation, the H-PSO effectively tracks MPP from 0.4 kW/m² to 1.0 kW/m² with minimal response time and stable power output. During load changes, the proposed tracker restores optimal power generation quickly with reduced oscillations compared to the PSO-P&O algorithm. The study verifies the H-PSO algorithm’s superior performance in simulation scenarios, but under certain conditions of rapid load and temperature variation, the algorithm’s inherent randomization nature limits its performance. This study shows the need for further development to improve robustness and adaptability under different conditions. Further work will include the development of more robust hybrid optimization algorithms and experimental validation of these algorithms with different PV configurations and real-world scenarios.

## 6. Future studies

Future work, based on the results of this research, will be directed to the further improvement of the H-PSO method with the goal of improving its performance under different working conditions, including high loading and varying temperatures. Moreover, the use of other hybrid optimization algorithms like Genetic Algorithm (GA) or Adaptive Neuro-Fuzzy Inference Systems (ANFIS) might enhance tracking efficacy and speed. Further, the practical applicability of the proposed algorithm will be tested in state-of-the-art laboratory settings with advanced PV systems using laboratory-scale HIL simulations. Moreover, the applicability of the H-PSO algorithm for larger-scale PV systems and the possibilities of its implementation with the smart grid infrastructure will be of great importance to improve energy management. Furthermore, by analyzing the effects of deploying H-PSO in large-scale renewable energy systems on the environment and the economy, the efficiency and affordability of the approach will be assessed.

## Supporting information

S1 Data Data in the experiment. This ZIP file contains simulation files, system configurations, and a Simulink model required to replicate the results. The model is compatible with MATLAB/Simulink R2024a.
(ZIP)
